# Absent Right Side Iliac Arterial System, an Intraoperative Surprise during Live Related Recipient Renal Transplantation

**DOI:** 10.1155/2015/894786

**Published:** 2015-08-11

**Authors:** Vijay Radhakrishnan, Rana Kumar, Datson George, G. P. Abraham

**Affiliations:** Department of Urology, PVS Memorial Hospital, Kaloor, Kerala 682017, India

## Abstract

*Introduction*. Renal transplantation has become the standard of care for patients with end stage renal disease. We present a rare case of an absent right sided iliac arterial system encountered during recipient renal transplantation. The presence of such vascular anomaly intraoperatively can present a technically challenging situation to the surgeon.* Case Presentation*. During a routine renal transplantation of a 34-year-old man, we encountered a complete absence of right side iliac arterial system and a prominent branch arising from left hemipelvis and coursing to the right lower limb and the urinary bladder. The artery to the bladder was divided and anastomosed end to end to the donor renal artery. Intraoperatively the renal perfusion and the urine output were good. A posttransplant magnetic resonance angiogram done six weeks later revealed good vascular supply to the kidney and the lower limb.* Conclusion*. Absent iliac artery on one or both sides is a rare phenomenon. The presence of it during an unanticipated renal transplant surgery can pose a significant technical challenge to the surgeons. We advocate routine assessment of pelvic vasculature before recipient renal transplant surgery so as to avoid a difficult situation like this.

## 1. Introduction

Renal transplantation surgery has become a standard of care in the management of ESRD. Usually donor kidney is placed in the right iliac fossa with anastamosis to right internal or external iliac artery and external iliac vein. We present a case of an absent/hypoplastic right sided iliac arterial system encountered intraoperatively during a routine recipient renal transplantation surgery. Absent iliac arterial system on one or both sides is a rare vascular anomaly with three cases [1–3] reported in the literature documenting the presence of the same in relation to recipient renal transplantation. The presence of such vascular anomaly could present a technically challenging situation to the transplant surgeon. We henceforth advocate a routine assessment of pelvic vasculature preoperatively to completely avoid such difficult situation.

## 2. Case Presentation

**Figure 1:**
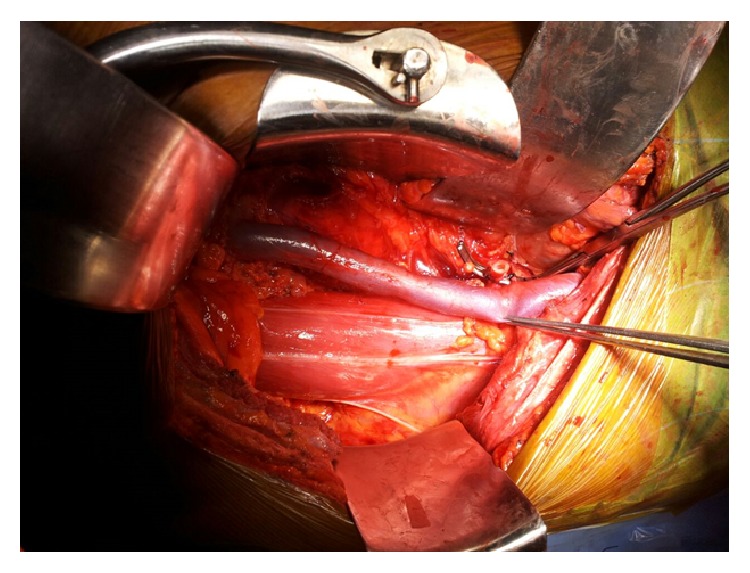
Intraoperative picture of right sided transplant fossa with complete absence of iliac arterial system.

**Figure 2:**
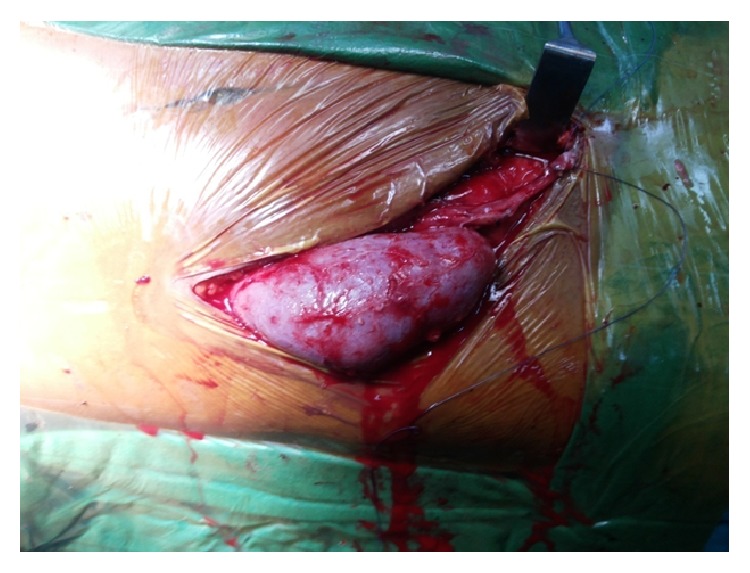
Postoperative image showing well-perfused transplanted kidneys.

**Figure 3:**
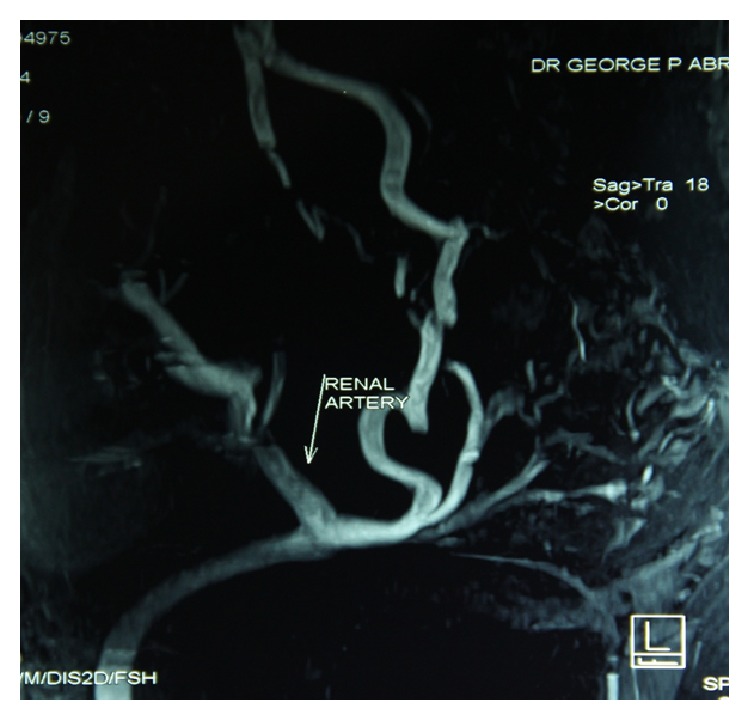
A MR angiogram done 6 weeks later revealed good vascular supply to the transplanted kidneys and the right lower limb. An absent or hypoplastic iliac system on the right side could as well be appreciated.

A 34-year-old patient with ESRD secondary to medical renal disease presented to us for renal transplant surgery. This patient apart from his renal failure did not present with any other symptom pertaining to other systems. The patient underwent all routine laboratory investigations and imaging as per the protocol including ultrasound abdomen and pelvis and a micturating cystogram (MCU). After appropriate written and informed consent, the patient was planned for renal transplant surgery. The patient was placed in supine position. Using an extended Gibson's incision, the right side retroperitoneum was approached. While preparing the transplant bed we encountered complete absence of right sided iliac arterial system, including common iliac (CIA), internal iliac (IIA), and external iliac arteries (EIA) (Figure 1). Distal dissection revealed the presence of an artery arising from the left hemipelvis, contributing a prominent branch to the urinary bladder and then making its way to the right sided lower limb. The artery to the bladder was divided and anastomosed end to end to the donor renal artery. The donor renal vein anastomosed end to side to the external iliac vein (EIV) as usual. Intra operatively the renal perfusion was good (Figure 2). The urine output was good. Ureteroneocystostomy was done using Taguchi's technique. Postoperative recovery was smooth. There was no evidence of limb ischemia. The followup of the patient was uneventful. A posttransplant magnetic resonance angiogram (MRA) was done 6 weeks later which revealed good vascular supply to the right kidney and the right lower limb (Figure 3).

## 3. Discussion

The presence of absent iliac arterial system on one or both sides is a very rare congenital anatomical entity [4] as compared to those involving the thoracic and abdominal aorta. The reported cases in the literature are very few.

A case of bilateral incidentally found aplasia of CIA [5, 6] has been described in the literature. A study of incidentally found bilateral EIA aplasia has been reported [7]. A case of bilateral IIA aplasia has been reported in a patient presenting with ruptured mycotic aneurysm of aorta [8].

The presence of unilateral vascular anomaly is more common as compared to the bilateral one. This anomaly has been reported more on the right side as compared to the left. Cases of unilateral vascular anomalies of EIA have been reported presenting asymptomatically [9] or with clinical evidence of ischemia of the lower limb [10–13]. Two cases of incidentally found absent CIA on the right side have been reported [14, 15] in the literature. The presence of vascular anomaly involving both iliac artery and vein has also been reported [9]. A case of abnormal left sided CIA and EIA with absent infrarenal inferior vena cava (IVC) with the presence of multiple congenital anomalies has been reported [16].

The most common difficulty encountered during recipient renal transplantation surgery arises from acquired causes like thrombosis of external iliac vein (EIV) and common iliac vein (CIV) and atherosclerotic stenosis or occlusion of IIA or CIA of iliac artery [17]. Congenitally absent iliac artery system in relation to renal transplantation is very rare with only three cases reported in the literature so far. Al Midani et al. [1] reported a case of absent right CIA found incidentally during workup of a live related transplantation. Tay et al. [2] reported a case of absent right CIA and EIA with congenital renal and genitor-urinary abnormality found preoperatively during routine imaging. Palkhi et al. [3] reported a case of complete absence of iliac arterial system on the left side diagnosed preoperatively in a case ESRD with a prior renal transplant surgery done on the right side.

Our case is unique in a way that the index patient did not have any other problem apart from the ESRD. He did not present with any symptoms including claudication of the limb or pelvic pain or any congenital renal and genitourinary problem. The patient underwent routine preoperative imaging protocol since the presence of any vascular anomaly could not be anticipated. To our surprise, we discovered intraoperatively a complete absence iliac arterial system on the right side.

## 4. Conclusion 

Absent iliac arterial system on one or both sides is a very rare phenomenon. The presence of it during an unanticipated routine renal transplant surgery can pose a significant technical challenge to the surgeons. We advocate routine assessment of pelvic vasculature before recipient renal transplant surgery in order to avoid a difficult situation intraoperatively.

## Abbreviations

ESRD: End stage renal disease
MCU: Micturating cystogram
CIA: Common iliac artery
IIA: Internal iliac artery
EIA: External iliac artery
EIV: External iliac vein
MRA: Magnetic resonance angiogram
IVC: Inferior vena cava
EIV: External iliac vein
CIV: Common iliac vein.
